# Virtual Reality Games as an Adjunct in Improving Upper Limb Function and General Health among Stroke Survivors

**DOI:** 10.3390/ijerph16245144

**Published:** 2019-12-16

**Authors:** Mohd Azzuan Ahmad, Devinder Kaur Ajit Singh, Nor Azlin Mohd Nordin, Khor Hooi Nee, Norliza Ibrahim

**Affiliations:** 1 Physiotherapy Programme, Centre for Rehabilitation and Special Needs, Faculty of Health Sciences, Jalan Raja Muda Abdul Aziz, Universiti Kebangsaan Malaysia, Kuala Lumpur 50300, Malaysia; azzuanahmad@ukm.edu.my (M.A.A.); norazlin8@gmail.com (N.A.M.N.); khorhn90@gmail.com (K.H.N.); 2 Physiotherapy Programme, Center for Healthy Ageing & Wellness, Faculty of Health Sciences, Jalan Raja Muda Abdul Aziz, Universiti Kebangsaan Malaysia, Kuala Lumpur 50300, Malaysia; 3 Physiotherapy Unit, Department of Medical Rehabilitation Services, Hospital Canselor Tuanku Muhriz, Kuala Lumpur 56000, Malaysia; lizaphysioppukm@gmail.com

**Keywords:** physiotherapy, virtual reality games, upper limb, function, stroke

## Abstract

Virtual reality (VR) games has the potential to improve patient outcomes in stroke rehabilitation. However, there is limited information on VR games as an adjunct to standard physiotherapy in improving upper limb function. This study involved 36 participants in both experimental (n = 18) and control (n = 18) groups with a mean age (SD) of 57 (8.20) and 63 (10.54) years, respectively. Outcome measures were the Fugl-Meyer assessment for upper extremities (FMA-UE), Wolf motor function test (WMFT), intrinsic motivation inventory (IMI), Lawton of instrumental activities of daily living (IADL), and stroke impact scale (SIS) assessed at pre-post intervention. The experimental group had 0.5 h of upper limb (UL) VR games with 1.5 h of standard physiotherapy, and the control group received 2 h of standard physiotherapy. The intervention for both groups was performed once a week for eight consecutive weeks. The results showed a significant time–group interaction effect for IMI (*p* = 0.001), Lawton IADL (*p* = 0.01) and SIS domain of communication (*p* = 0.03). A significant time effect was found in FMA-UE (*p* = 0.001), WMFT (*p* = 0.001), Lawton IADL (*p* = 0.01), and SIS domains; strength, ADL and stroke recovery (*p* < 0.05). These results indicated an improvement in UL motor ability, sensory function, instrumental ADL, and quality of life in both groups after eight weeks of intervention. However, no significant (*p* > 0.05) group effect on all the outcome measures was demonstrated. Thus, replacing a portion of standard physiotherapy time with VR games was equally effective in improving UL function and general health compared to receiving only standard physiotherapy among stroke survivors.

## 1. Introduction

Stroke is a leading cause of significant disability among adults globally [1]. Rehabilitation is of utmost importance with an increase in the number of stroke survivors [2]. Stroke rehabilitation requires a multidisciplinary approach, is long-term and challenging due to its complexity [3]. Recent evidence suggests that the extension of a stroke rehabilitation programme may lead to further improvement in function and quality of life among stroke survivors [3].

Persistent upper limb (UL) dysfunction after a stroke is one of the most challenging issues in rehabilitation [4]. Increasing the dose of rehabilitation among stroke survivors may improve outcomes, and one of the strategies includes performing self-administered exercises using VR games technology [4]. VR is a computer-assisted technology that can provide users with experiences of a simulated “real” environment [5]. VR technology has been used in rehabilitation in addition to standard physiotherapy, or as a preventive therapy [5]. VR-based rehabilitation also offers the capacity to individualise treatment needs while providing the standardisation of assessment and training protocols [5].

Earlier evidence suggested that VR technology can provide a unique medium whereby rehabilitation can be delivered in a functional and purposeful manner [6]. Moreover, VR technology-based rehabilitation can be readily graded and documented [6]. Other than that, stroke survivors can perform VR training at their home and the therapist can monitor from a distance, known as tele-rehabilitation [7]. Compliance towards treatment and rehabilitation is a vital factor to consider in stroke management [8]. Hence, VR rehabilitation has the potential to improve patient participation, enable intensive therapy and reduce demand on health care professionals [5,6,7]. 

In previous studies, VR games were shown to be effective in improving physical function among stroke survivors [9], balance and functional mobility in older adults [10,11], and upper limb reaction time in adults with physical disabilities [12]. However, balance and mobility issues were examined rather than upper limb function [9,11]. There is also limited information on VR games as an adjunct to standard physiotherapy. Moreover, previous evidence mainly demonstrates the effects of VR as a standalone intervention among stroke survivors [13,14]. For example, in a pilot crossover design study involving 14 participants with chronic stroke, VR game-assisted intervention was performed for 45–60 min for a duration of 2.5 weeks [14]. The results showed improved UL motor performance using the Fugl-Meyer assessment for upper extremities (FMA-UE) as the primary outcome measure. In our present study, we aimed to examine the effectiveness of VR games as an adjunct to standard physiotherapy in improving upper limb (UL) function and general health among stroke survivors.

## 2. Materials and Methods

### 2.1. Participants

The required sample size was calculated using the G-Power analysis program version 3.1.9.2 [15]. A total of 34 participants was suggested to observe a significant difference between two different groups within effect size 0.25, significance level *p* < 0.05, and power 0.80. Forty participants were recruited from Hospital Canselor Tuanku Muhriz (HCTM) in this clinical trial. The inclusion criteria were: (1) stroke survivors at least 6 months post-stroke to include those with chronic stroke, (2) aged 18 years and above, (3) the affected arm scored at least 4 out of 6 according to the motor assessment scale, and (4) able to participate in the VR games training without limitation (presently with good health and no self-reported orthopaedic, medical, or painful conditions). Those with severe cognitive impairments (MMSE score less than 17) and taking any prescribed drugs that could potentially affect physical function and balance (such as corticosteroids, antipsychotics, or antidepressants) were excluded from the study.

Trained research assistants assisted by a physiotherapist from HCTM performed screening, the selection of participants, and supervised VR games training. Participants were informed about the study protocol and they signed a written informed consent before the start of the intervention. The study was approved by the Research and Ethics Committee of Universiti Kebangsaan Malaysia (UKM1.5.3.5/244/NN-110-2012) and registered with the Australian New Zealand Clinical Trials Registry (ANZCTR: ACTRN12618000725268).

### 2.2. Procedures

Forty eligible participants were allocated into two equal-size groups (experimental and control) by simple randomization. Four participants, two from each group, withdrew from the study due to unavoidable personal circumstances. Outcome measures were evaluated before and immediately on completion of the 8 weeks of intervention. The experimental group (n = 18) had 0.5 h of VR games using Cy-Wee Z game controller and another 1.5 h of standard physiotherapy exercises. The control group (n = 18) continued with their 2 h of routine standard exercise therapy supervised by a physiotherapist. Each session comprised of stretching, strengthening, gait training, coordination, balance, and functional exercises, such as sit to stand and stair climbing. Both groups received eight therapy sessions, one per week for eight continuous weeks. An independent assessor carried out the measurement of all outcome measures at baseline (week 0) and eight weeks of intervention using standardised tools.

### 2.3. Virtual Reality Games 

In addition to 1.5 h of standard physiotherapy exercises, participants in the experimental group had VR games intervention for 0.5 h in the sitting position, supervised by a therapist. The single-user VR games comprised of a computer with 21 inches monitor and a Cy-Wee Z movement-based game controller. This game controller is equipped with accelerometer, gyroscope and magnetic sensors which enables display of free movement in 3-dimensional space and capacity to detect depth. A custom-made handlebar was incorporated into the Cy-Wee Z game controller to encourage bilateral upper limb movements.

The games selected consisted of various physical challenges such as stationary and moving target-hitting and sports related games. Examples of the games includes Mosquito Swat, Music Catch, ReBounce, Bejewelled, and Balloon Popping, 10-Pin Bowling, Air Hockey, Mah-Jong, and Solitaire. All of these games required large cursor movements in both horizontal and vertical directions to facilitate movements of the affected UL. Progress in the difficulty levels of the games was adjusted according to participants’ individual achievements. Several measures were taken to ensure participants engagements during the VR intervention. The VR games intervention was conducted in a room with adequate space to avoid distraction from surroundings and allow unrestricted movements. An adjustable sound speaker was used for clear auditory feedback. The distance between the chair and monitor display was adjusted according to individual participants’ preferences.

### 2.4. Outcome Measures

The Fugl-Meyer assessment for upper extremities (FMA-UE) is a measure of UL motor and sensory impairment. FMA-UE commonly used in clinical and research and is one of the most common quantitative measures of motor impairment among stroke survivors [16,17]. Intra-rater reliability for the expert rater was high for the motor and sensory scores with a range of 0.95 to 1.0 [18].

The Wolf motor function test (WMFT) is a quantitative measure of UL motor ability, through timed and functional tasks, specifically for the evaluation of chronic stroke and traumatic brain injury patients [19]. The psychometric properties of the WMFT is excellent with an ICC score of 0.94 [20].

Intrinsic motivation inventory (IMI) is a multidimensional questionnaire to assess participants’ subjective experiences related to activity [21]. IMI has been widely used in several types of research to evaluate the intrinsic motivation and self-regulation of participants [21]. The reliability (Cronbach’s alpha) for IMI as a whole was 0.844 [22].

The Lawton of instrumental activities of daily living (IADL) scale assesses the more complex ADLs necessary for living in the community. There are eight domains of function measured by the instrument: an ability to use a telephone, shopping, food preparation, housekeeping, laundry, mode of transportation, responsibility for own medications, and the ability to handle finances [23]. Inter-rater reliability of Lawton IADL was 0.85 [23].

The stroke impact scale (SIS) is a stroke-specific and self-report questionnaire designed to evaluate disability and quality of life (QOL) after stroke [24]. The internal consistency of the SIS is excellent with Cronbach’s alpha values ranging from 0.80 to 0.95 [24].

### 2.5. Statistical Analysis

Data were analysed using Statistic Product for Statistical Solutions (SPSS) version 19.0 (SPSS Inc. Chicago, IL, USA). ANCOVA and Chi-Square tests were used to tests the effect of age, post-stroke duration, and the affected side of stroke as dependent variables. The main effects of time, group, and time–group interaction were analysed using repeated measure ANOVA. The significance level was set at *p* < 0.05.

## 3. Results

**Figure 1:**
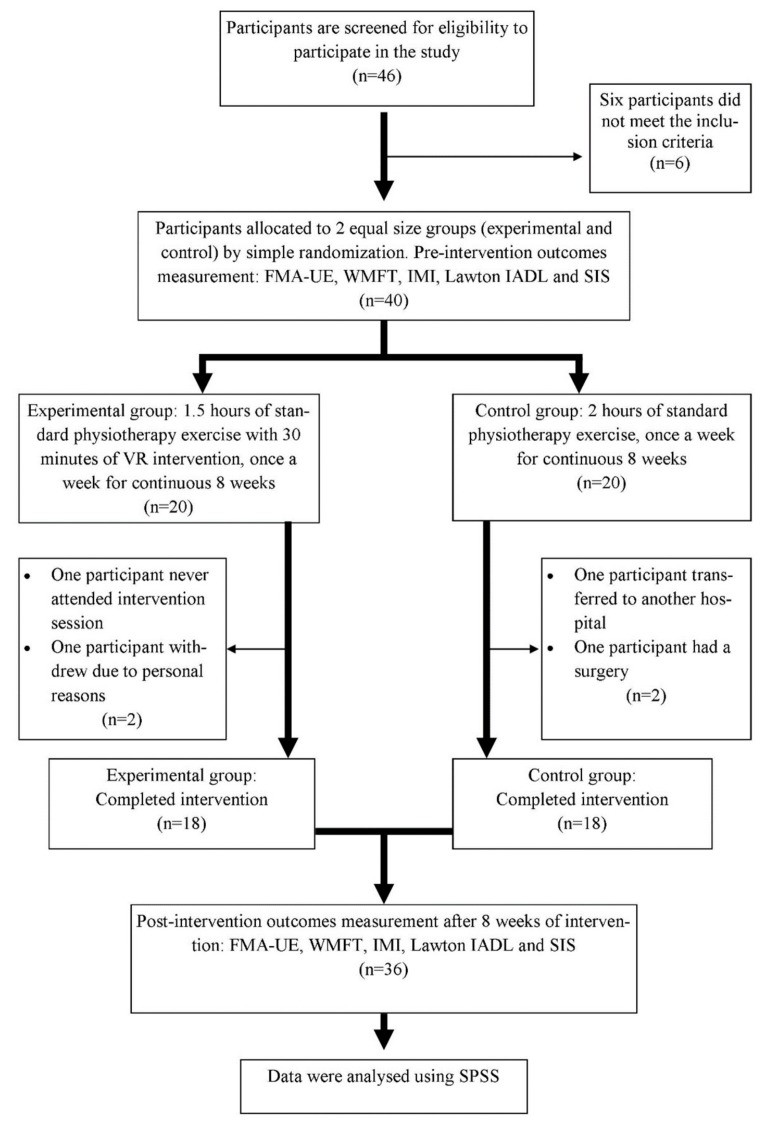
Flow chart of the study with number of participants. FMA-UE, Fugl-Meyer assessment for upper extremities; WMFT, Wolf motor function test; IMI, intrinsic motivation inventory; IADL, instrumental activities of daily living; SIS, stroke impact scale; VR, virtual reality; SPSS, statistic product for statistical solutions.

Thirty-six participants (18 experimental and 18 control) completed the study. Figure 1 shows the flow of the participants through the study. Participants’ demographic data at baseline are presented in Table 1. ANCOVA and Chi-Square tests demonstrated that there was no significant effect of age, post-stroke duration, and the affected side of stroke as dependent variables. The results of all outcome measures at baseline and eight weeks post-intervention are listed in Table 2. Repeated measure ANOVA showed a significant effect of time in FMA-UE (*p* = 0.001; ηp2 = 0.76), WMFT (*p* = 0.001; ηp2 = 0.79), Lawton IADL (*p* = 0.01; ηp2 = 0.17), and the SIS domains of strength (*p* < 0.001; ηp2 = 0.32), ADL (*p* = 0.001; ηp2 = 0.24), and stroke recovery (*p* = 0.001; ηp2 = 0.56). However, no significant effect of group on all the outcome measures was found. The results indicate that both groups (experimental and control) improved UL physical function (motor and sensory), instrumental ADL, and QOL after eight weeks of intervention. 

**Figure 2:**
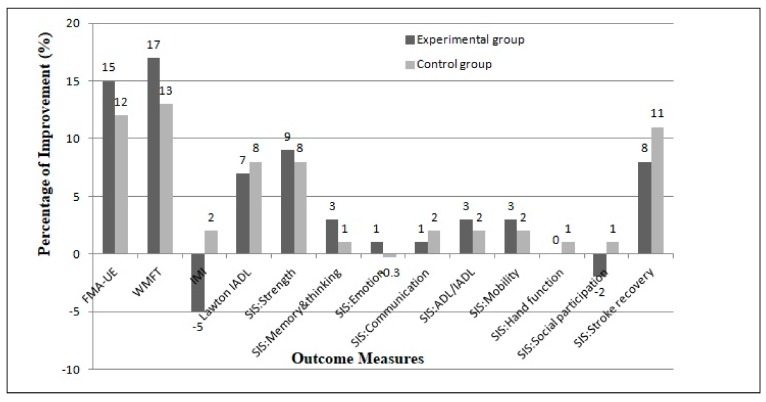
Percentage of improvement for the outcome measures after eight weeks of intervention. FMA-UE, Fugl-Meyer assessment for upper extremities; WMFT, Wolf motor function test; IMI, intrinsic motivation inventory; IADL, instrumental activities of daily living; SIS, stroke impact scale.

Higher percentages of improvement were demonstrated in the experimental group compared to the control group for FMA-UE, WMFT, and several SIS domains, namely strength, memory and thinking, emotion, ADL/IADL, and mobility (Figure 2). Interestingly, there is no significant difference between the two interventions as shown by the main effect of group. This suggests that the experimental and control groups had similar outcomes after the intervention. Despite no overall difference between the two groups, the analysis of time–group interaction showed significant effects for IMI (*p* = 0.001; ηp2 0.20), Lawton IADL (*p* = 0.01; ηp2 = 0.19), and the SIS domain of communication (*p* = 0.03; ηp2 = 0.13). These results indicate that the time course for the two intervention is significantly different for the three outcomes. The control group showed slightly more improvements in instrumental ADL, communication, and higher self-perceived positive experience measured using IMI (Figure 2).

## 4. Discussion

The objective of this study was to examine the effectiveness of VR games as an adjunct to standard physiotherapy in improving UL function and general health among stroke survivors. The results indicated that participants in both groups improved in their UL motor ability, sensory function, instrumental ADL, and quality of life after eight weeks of intervention.

Higher percentages of improvement were observed in the experimental group (added VR games to standard physiotherapy) compared to the control (only standard physiotherapy) group for FMA-UE, WMFT, and SIS domains of strength, emotion, ADL, mobility, memory, and thinking (Figure 2). However, no statistically significant difference between the two groups was found in all the outcome measures. These findings suggest that participants in the experimental group did not improve more than those in the control, and both interventions were equivalent in terms of effectiveness. This result supports the substitution of a portion of standard physiotherapy time with VR games among stroke survivors. It is noteworthy that VR games may be self-administered by patients, thus freeing therapist time for managing patients with more acute problems.

Although both groups demonstrated similar outcomes in improving UL motor ability and sensory function, the experimental group showed a higher percentage of improvements (FMA-UE 15%, WMFT 17%) compared to the control (FMA-UE 12%, WMFT 13%). This is consistent with previous studies that showed improvements in UL motor and sensory function among stroke survivors following VR training as measured using FMA-UE and WMFT [14,25,26]. We used a similar model of the game controller for VR games intervention as in the pilot study by Hijmans et al. [14]. The strength of our pre-post study with a matched control at baseline is the use of VR games as a substitution for a portion of standard physiotherapy treatment instead of using VR games on their own.

Similarly, Levin et al. [26] stated that two-dimensional video-capture VR training led to a significant improvement in WMFT scores compared to standard physiotherapy. In our study, the improvements in both groups can be explained by the theory of neuroplasticity. In theory, the repetitive task-orientated practice can provide more effective motor relearning for neuronal recovery following stroke [5]. As for VR games training, intensive cursor movements involving bilateral hands in various directions when playing the games could have led to the additional effort that further enhances neuronal recovery [5,7]. In our present study, a custom-made handlebar was incorporated to facilitate motor movements of the affected hand by using the non-affected hand. A similar method has been used in a previous study where the affected hand was bandaged to the game controller if the participant was unable to hold it effectively to achieve bilateral hand activities [13].

In our study, the improvement in instrumental ADL could be due to the nature of VR games that provided a continuous challenge to increase UL coordination and reaction time. The VR games training also required various repeated movements that resembled UL functional activities. Our study findings were in agreement with a previous study, which showed a significant improvement in instrumental ADL using a VR shopping task [27]. Improvement in instrumental ADL is vital in achieving independent living and an improved QOL among stroke survivors.

As for the improvement in SIS domain of communication with VR games, a similar finding was found using VR training for 30 min followed by 15 min of functional training such as using a phone and cutting with a knife [28]. Communication improvements among stroke survivors in our study could have been due to the indirect effect of engagement and immersion via VR games. However, further studies are required to explore the use of single and multi-user VR training in improving communication among stroke survivors.

Despite the unique attributes of VR games, which include fun and dynamic training [13,14], participants in the experiment showed a reduction in IMI score after eight weeks of the intervention (Figure 2). This finding was unexpected and suggests that participants in the experiment preferred standard physiotherapy rather than VR Cy-Wee Z training. In contrast to Hale et al. [13], the perceptions of stroke survivors were acceptable and potentially beneficial when using a VR games Cy-Wee Z game controller for UL rehabilitation. Generally, VR gaming-based rehabilitation was reported to be highly accepted with overall satisfaction among stroke survivors and healthy controls [29]. Among the possible reasons for our contradictory results may be the lack of availability of diverse and interesting games in VR Cy-Wee Z and participants who were contented with their current therapy. Further, we used an individual-based questionnaire compared to an in-depth interview and open-ended focus group discussions, which could have provided more insights about participants’ perceptions [13].

One limitation of our study is related to the quasi-experimental design that limited the randomization of participants. However, both experimental and control groups were similar in terms of their demographic data at baseline. The short duration of VR games intervention (0.5 h of VR games once a week for eight weeks) may be considered as inadequate, and this could be one of the reasons for the non-significant effects of adding VR games to standard physiotherapy. Moreover, potential confounding factors, such as baseline stroke severity and subtypes, were not taken into account in our study as this information was not available. Further qualitative and quantitative research with a larger sample size and longer duration of intervention may be required to determine the effectiveness of VR games for UL rehabilitation among stroke survivors.

## 5. Conclusions

The integration of VR games as an adjunct to standard physiotherapy for UL stroke rehabilitation was demonstrated to be equally beneficial compared to standard physiotherapy on its own. The use of VR games in our study was not intended to replace the current rehabilitation. On the other hand, VR games exercise can be used by patients at home to increase the dosage of exercise and self-management, reduce healthcare dependency, and as an adjunct to aid therapists in the challenging process of stroke rehabilitation.

## Figures and Tables

**Table 1:** Baseline characteristics of participants.

Variable	Experimental (*n* = 18)	Control (*n* = 18)	Analysis of Covariance
	Mean (SD)	Mean (SD)	*p*-Values
Gender (male/female)	17/1	14/4	
Age (years)	57.00 (8.203)	62.94 (10.54)	0.07
Post stroke duration (months)	10.56 (5.26)	10.72 (6.03)	0.93
Affected side (left/right)	11/7	9/9	0.50

All data as mean (standard deviation) with a *p*-value for ANCOVA except for affected side (left/right) with *p*-value for Chi-squared test. Statistically significant, *p* < 0.05.

**Table 2:** Main effect of time, group, and time–group interaction of the interventions on the outcome measures.

Parameters	Study Group		Analysis of Covariance (*p*-Values)		
	Experimental Mean (SD)	Control Mean (SD)	Time (ηp^2^)	Group (ηp^2^)	Interaction (ηp^2^)
**FMA-UE**					
Week 0	57.44 (10.17)	56.50 (8.18)	0.001 * (0.76)	0.50 (0.01)	0.23 (0.04)
Week 8	65.94 (7.57)	63.22 (6.98)			
**WMFT**					
Week 0	46.00 (10.99)	44.11 (7.64)	0.001 * (0.79)	0.38 (0.02)	0.17 (0.05)
Week 8	53.61 (10.35)	50.05 (7.55)			
**IMI**					
Week 0	126.94 (11.40)	118.22 (8.35)	0.16 (0.06)	0.14 (0.06)	0.001 * (0.20)
Week 8	120.50 (10.00)	120.50 (8.37)			
**Lawton IADL**					
Week 0	4.28 (2.22)	2.78 (1.00)	0.01 * (0.17)	0.77 (0.00)	0.01 * (0.19)
Week 8	4.56 (1.98)	3.00 (1.03)			
**SIS domains**					
**Strength**			0.00 * (0.32)	1.00 (0.00)	0.16 (0.06)
Week 0	57.29 (15.93)	63.54 (12.91)			
Week 8	62.50 (11.94)	68.75 (13.89)			
**Memory, thinking**			0.05 (0.11)	0.34 (0.03)	0.21 (0.05)
Week 0	87.91 (14.24)	82.94 (11.39)			
Week 8	90.96 (11.39)	84.02 (15.32)			
**Emotion**			0.78 (0.00)	0.57 (0.01)	0.34 (0.30)
Week 0	83.97 (12.92)	80.40 (13.46)			
Week 8	84.79 (12.29)	80.13 (13.37)			
**Communication**			0.13 (0.07)	0.51 (0.01)	0.03 * (0.13)
Week 0	94.84 (8.85)	84.92 (17.70)			
Week 8	95.64 (9.05)	86.91 (14.13)			
**ADL/IADL**					
Week 0	84.32 (12.05)	77.41 (11.11)	0.001 * (0.24)	0.67 (0.00)	0.06 (0.10)
Week 8	86.68 (10.31)	79.23 (11.11)			
**Mobility**					
Week 0	85.34 (10.52)	80.86 (15.06)	0.06 (0.10)	0.82 (0.00)	0.25 (0.04)
Week 8	87.50 (6.95)	82.56 (15.12)			
**Hand function**					
Week 0	68.61 (27.64)	66.11 (22.59)	0.77 (0.00)	0.77 (0.00)	0.79 (0.00)
Week 8	68.61 (27.80)	66.67 (22.10)			
**Social**					
Week 0	74.31 (16.31)	68.58 (18.44)	0.74 (0.00)	0.19 (0.05)	0.45 (0.02)
Week 8	72.63 (17.95)	69.59 (16.47)			
**Stroke recovery**					
Week 0	68.61 (12.70)	61.67 (9.85)	0.001 * (0.56)	0.45 (0.02)	0.09 (0.08)
Week 8	73.89 (10.37)	68.33 (11.38)			

Values are presented as mean ± standard deviation. ηp^2^, partial eta squared; FMA-UE, Fugl-Meyer assessment for upper extremities; WMFT, Wolf motor function test; IMI, intrinsic motivation inventory; IADL, instrumental activities of daily living; SIS, stroke impact scale. Statistically significant, * *p* < 0.05 by repeated measure ANOVA.
